# ‘Screening audit’ as a quality assurance tool in good clinical practice compliant research environments

**DOI:** 10.1186/s12910-018-0269-2

**Published:** 2018-04-25

**Authors:** Sinyoung Park, Chung Mo Nam, Sejung Park, Yang Hee Noh, Cho Rong Ahn, Wan Sun Yu, Bo Kyung Kim, Seung Min Kim, Jin Seok Kim, Sun Young Rha

**Affiliations:** 1 Human research Protection Center, Severance Hospital, Yonsei University Health System, Seoul, Korea; 2 Department of Preventive Medicine, Yonsei University College of Medicine, Seoul, Korea; 3 Department of Biostatistics, Yonsei University College of Medicine, Seoul, Korea; 4 Department of Neurology, Yonsei University College of Medicine, Seoul, Korea; 5 Division of Hematology, Department of Internal Medicine, Yonsei Cancer Center, Yonsei University College of Medicine, Seoul, Republic of Korea; 6 Division of Medical Oncology, Department of Internal Medicine, Yonsei Cancer Center, Yonsei University College of Medicine, 50-1 Yonsei-ro, Seodaemun-gu, Seoul, 03722 Korea

**Keywords:** Screening audit, Clinical research, Quality assurance

## Abstract

**Background:**

With the growing amount of clinical research, regulations and research ethics are becoming more stringent. This trend introduces a need for quality assurance measures for ensuring adherence to research ethics and human research protection beyond Institutional Review Board approval. Audits, one of the most effective tools for assessing quality assurance, are measures used to evaluate Good Clinical Practice (GCP) and protocol compliance in clinical research. However, they are laborious, time consuming, and require expertise. Therefore, we developed a simple auditing process (a screening audit) and evaluated its feasibility and effectiveness.

**Methods:**

The screening audit was developed using a routine audit checklist based on the Severance Hospital’s Human Research Protection Program policies and procedures. The measure includes 20 questions, and results are summarized in five categories of audit findings. We analyzed 462 studies that were reviewed by the Severance Hospital Human Research Protection Center between 2013 and 2017. We retrospectively analyzed research characteristics, reply rate, audit findings, associated factors and post-screening audit compliance, etc.

**Results:**

Investigator reply rates gradually increased, except for the first year (73% → 26% → 53% → 49% → 55%). The studies were graded as “critical,” “major,” “minor,” and “not a finding” (11.9, 39.0, 42.9, and 6.3%, respectively), based on findings and number of deficiencies. The auditors’ decisions showed fair agreement with weighted kappa values of 0.316, 0.339, and 0.373. Low-risk level studies, single center studies, and non-phase clinical research showed more prevalent frequencies of being “major” or “critical” (*p* = 0.002, < 0.0001, < 0.0001, respectively). Inappropriateness of documents, failure to obtain informed consent, inappropriateness of informed consent process, and failure to protect participants’ personal information were associated with higher audit grade (*p* < 0.0001, *p* = 0.0001, *p* < 0.0001, *p* = 0.003). We were able to observe critical GCP violations in the routine internal audit results of post-screening audit compliance checks in “non-responding” and “critical” studies upon applying the screening audit.

**Conclusions:**

Our screening audit is a simple and effective way to assess overall GCP compliance by institutions and to ensure medical ethics. The tool also provides useful selection criteria for conducting routine audits.

## Background

Good Clinical Practice (GCP) is an international scientific and ethical quality standard for designing, conducting, recording, and reporting clinical research using human participants. It originated from the Declaration of Helsinki [1]. Clinical research using human participants should ensure GCP compliance throughout the research period. Investigators are responsible for ensuring that the research activities achieve initial or continuing approval from the relevant Institutional Review Board (IRB). In addition, an independent method for confirming whether research complies with the relevant laws and regulations is an audit. An audit is a systematic monitoring method used to determine whether clinical-research-related activities and documents are adequately obtained according to the approved protocol, GCP, and the applicable regulatory requirements.

In recent years, the scope and amount of clinical research has expanded, and regulations are becoming more stringent [2, 3]. Increasingly, human research protection programs (HRPPs) are being introduced by research organizations to promote ethical values in the research community and to ensure an environment of participant protection. As a component of HRPP, an audit confirms all research documents and procedures in regards to whether they follow regulations and protocol. Auditing procedures, however, are not easy for most organizations, and encompass preparing an audit’s standard of procedure (SOP), protocol selection, recording the audit trail, source document verification, and investigator interviews, which require approximately three to 4 days and expert staff. Furthermore, an internal audit cannot cover all active protocols because of time limits. Accordingly, audits are time consuming and can be a laborious burden for both organizations initially adopting an HRPP and organizations already operating one. Seeing an unmet need for measures with which to ensure quality compliance by research organizations, we developed a “screening audit” that confirms whether trial documents have been properly set aside and whether all active protocols are being adhered to: it does this without interviews or source document verification. The aim of this study was to evaluate the effectiveness of the screening audit in confirming GCP compliance status among investigators and research institutions.

## Methods

### Development of the screening audit

**Fig. 1:**
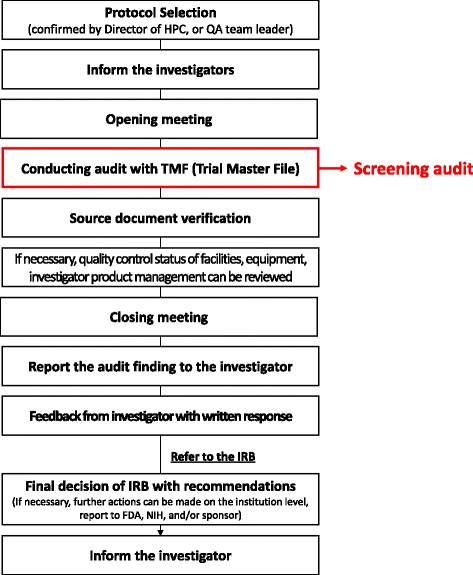
Scheme of detailed process of a routine audit and the scope of a ‘screening audit’

This study was conducted as a preliminary exploratory study through which to introduce the screening audit and to provide information on its effectiveness based on data from a single institution. The scope of an audit can vary according to the institution or sponsor’s SOP. While routine audit is a scrutinizing way to confirm GCP compliance status, it is also laborious and time consuming. A detailed routine audit consists of 10 steps, and the screening audit is applicable to the step of trial master file (TMF) review (Fig. 1). At the opening meeting, the auditor explains the target, scope, and purpose of the audit, and may ask the investigator to give an overall explanation about the protocol or GCP in order to evaluate the investigator’s understanding thereof. The primary areas of concern are the availability of essential documents and the appropriateness and completeness of documentation.

We developed the screening audit based on the internal audit procedure of Severance Hospital. The screening audit is performed only with available documents, without interviews, response feedback, or data accuracy evaluations, as shown in Table 1. The checklist was composed of 20 questions grouped into five audit-finding categories (Table 2). Instead of an IRB decision on the audit findings and response, auditors themselves make a decision on each protocol based on the findings from the screening audit. Both routine audit and screening audit should follow the ICH-GCP [1], U.S. Food and Drug Administration (FDA) guidance [4], and Korean GCP.

**Table 1:** Comparison of key components between routine audit and screening audit

Item	Routine audit	Screening audit
Target protocols	Active protocols approved by IRB	Active protocols approved by IRB
Protocol Selection	1–2 protocols per week upon the risk matter (i.e. Early phase study, protocols with vulnerable participants, etc.)	Every active protocols at the time when the screening audit conducted
Initiation meeting with investigators	About 30 min	Not required
Details of review		
1) Regulatory documentation (i.e., IRB/FDA approval letter, Contract, Protocol, etc.)	- Availability - Accuracy and completeness	- Availability
2) Participant logs	- Availability - Accuracy and completeness	- Availability
3) Investigator’s log and CV	- Availability - Qualification - Scope of the delegation	- Availability
4) Consent process	- Availability of obtained ICF - Adequacy of consent process	- Availability of obtained ICF - Adequacy of consent process
5) Protocol compliance	- Source documents and medical records verification	- Not required
6) Case Report Forms	- Availability - Accuracy and completeness	- Availability
7) Reports	- Safety issues detection and report - Compliance for report to sponsor/IRB/FDA	- Availability of documentation
8) Pharmacy, laboratory, other facility maintenance	- Availability of quality control record - Facility check	- Availability of quality control record
9) Specimen maintenance	- Availability of related record - Accuracy and completeness of maintenance log - Facility check	- Availability of documentation
Closing meeting with investigators	About 30 min	Not required
Reporting the audit findings	Inform the investigators of the findings Response of investigators is needed	Inform the investigators of the findings Response is not required
Decision	Decision made by IRB in the convened meeting with audit findings and investigator’s response	Decision made by auditors with only audit findings
Follow up measure	Follow up with investigator’s corrective action and preventive action	Recommend full routine audit or for-cause audit

*CV* curriculam vitae, *ICF* informed consent form

**Table 2:** Screening audit checklist derived by Severance Hospital

Questions	Yes/No	If no, it counts on the following category
Study team has secured and kept a delegation log. (e.g., name, duration of study participation, role, signature, etc.).		Failure to maintain essential documents
Study team has been trained according to the HRPP P&P and its certificate are maintained with their CV.		
Study team maintains relevant documents with IRB such as IRB application, IRB approval letter, and IRB roaster.		
Study team maintains documents such as protocol, case report form, informed consent form, etc. submitted to the IRB with all revised versions.		
Study team maintains participant logs; Participant screening log, Enrollment log, Randomization code (if, applicable), Identification log.		
Study team maintains completed case report form of each enrolled participant.		
All items that the investigators must fill out are completed.		Inappropriateness of documents
No errors were discovered when browse through the documents.		
Study team has followed CRF guidelines regarding documentation and correction.		
PI signed in the completed CRF.		
Prior to participation in clinical research, subjects (or their LAR) wrote his/her hand written signature and date on the consent form (including subjects who failed screening test or their LAR)		Failure to obtain informed consent
In clinical research with human materials, subjects also wrote his/her hand written signature and date on the statutory consent form from the Bioethics and Safety Act.		
Principle Investigator or designated and qualified research staff obtained ICF from the subjects.		
[Witness] In case of the person who cannot read the document (or LAR), the witness attended the consent process, and signed in the consent form while the subject (or LAR) agreed with verbal consent.		
[Legally Authorized Representative] In case of the person who lacked understanding or expression ability, both subjects and their legally authorized representative signed the consent form.		
All items that the subject must fill out are completed including optional check.		Inappropriateness of informed consent form
No errors were discovered when browse through the documents.		
Re-consent has been made in case of ICF modification.		
Study team protect subject’s personal information with identification code.		Failure to protect participants’ personal information
Identifiable data (name, hospital number, social number, etc.) is not written on the CRF.		

Screening audit was performed annually for all ongoing clinical research, including clinical trials with investigational drugs/devices, biospecimen research, social behavioral research, etc. The relevant IRB should determine a protocol’s risk level (out of four possible grades) based on a risk and benefit analysis. Risk level 1 is minimal risk, which is encountered in daily life or at a routine doctor’s visit. Risk level 2 means minor increase over minimal risk, and risk level 3 goes to possibility of significant sequelae, must be transient. Risk level 4 is defined when the research subjects could experience death or irreversible, debilitating damage, or major unknowns with respect to the intervention.

Audit findings were presented as a numeric count. Auditors classified findings into four different grades, including ‘Not a finding’, ‘Minor’, ‘Major’, and ‘Critical’. According to Korea FDA guideline and Severance Hospital Human Research Protection Program Policy and Procedure (HRPP P&P), ‘not a finding’ means a protocol in which there are no findings, while ‘Minor’ decision was made when complementary measures are necessary to ensure participant safety and reliability of the research ethics. ‘Major’ is defined as findings that are likely to put a participant at risk or to hinder the quality of the research, while ‘Critical’ is defined as findings significantly affecting the reliability of the research results, especially when major findings are continuously or repeatedly identified in the routine audit. In case of screening audit, protocols with more than three missing documents or inappropriate consent processes or failure to obtain informed consent forms were defined as ‘Critical’; more than two missing documents was defined as ‘Major’; and one missing documents was defined as ‘Minor’. Finally, protocols in which no problems are detected are defined as ‘Not a finding’.

### Materials

We retrospectively reviewed the screening audit records of 462 protocols regularly assessed by the Human Research Protection Center at Severance Hospital, Yonsei University College of Medicine, Seoul, Korea, between January 2013 and January 2017. Protocol inclusion criteria for the screening audit were all active protocols approved by Severance Hospital’s IRB, with greater focus on investigator-initiated trials, prospective studies, and the protocols that have not undergone routine audits. An IRB was established in Severance Hospital in 1997, and all active protocols approved by the IRB since its establishment were included. The screening audit was conducted by five qualified auditors with more than 2 years of experience in the Human Research Protection Center quality assurance (QA) team. In order to evaluate the validity of the audit results, the auditors cross-checked each other’s audit findings with blind study information and made an assessment of their own. An assessment of the results’ validity was conducted with 265 protocols between January 2013 and January 2014.

### Statistical method

All analyses were conducted using SAS version 9.4 (SAS Institute Inc., Cary, NC) or SPSS 23.0 for Windows (SPSS Inc., Chicago, IL), with a *p*-value < 0.05 being considered statistically significant. Descriptive data are expressed as a mean ± standard deviation and categorical variables as a frequency and percentage. Differences between screening audit results according to year, risk level, and department were analyzed with Chi-square test and Fisher’s exact test. Validation between results evaluated by three individual auditors were sought using the weighted kappa. Finally, a comparison of the audit findings among different grading groups or finding categories were conducted using the Chi-square test. To confirm the ordinal variation, Mantel-Haenszel Chi-square test was conducted.

## Results

### Baseline characteristics of protocols

Table 3 shows the basic characteristics of the protocols that underwent screening audit. Most protocols involved non-phase clinical research (*n* = 361, 78.1%), although we also covered phase 1 to 4 clinical trials. The majority exhibited minimal risk (*n* = 263, 56.9%), 152 (32.9%) were level 2, 31 (6.7%) were level 3, and one (0.2%) was level 4. The study determined to be risk level 4 was a prospective clinical study involving a surgical procedure for an aortic stenosis patient proposed by cardiology investigators, and the IRB analyzed the anticipated mortality risk in comparison with other standard surgical procedures. The prevalent characteristics of the studies were “conducted at a single center” (*n* = 281, 60.8%) and academic purpose (*n* = 455, 98.1%). Principle investigators who had experienced screening audits mainly came from the Department of Internal Medicine (*n* = 179, 38.7%).

**Table 3:** Baseline characteristics of protocols reviewed by screening audit

Characteristics	N(%)
Phase	
Phase 1, 1/2	3 (0.6)
Phase 2	36 (7.8)
Phase 2/3	2 (0.4)
Phase 3	13 (2.8)
Phase 4	40 (8.7)
Post-market surveillance	1 (0.2)
Medical device	6 (1.3)
Non-phase clinical research	361 (78.1)
Total	462 (100)
Multicenter	
Single center	281(60.8)
Domestic multicenter	162(35.1)
International multicenter	19(4.1)
Total	462 (100)
Department of principle investigator^c^	
Clinical department I	179(38.7)
Clinical department II	45(9.7)
Clinical department III	123(26.6)
Supportive departments	101(21.9)
Basic Science departments	14(3.0)
Total	462 (100)
Risk Level^a^	
Level I	263(56.9)
Level II	152(32.9)
Level III	31(6.7)
Level IV	1(0.2)
Undeterminated^b^	15(3.2)
Total	462 (100)
Responsible entity	
Investigator initiated	455(98.5)
Sponsor initiated	7(1.5)
Total	462 (100)

^a^Level 1=minimal risk, Level 2=minor increase over minimal risk, Level 3= Possible significant sequelae, must be transient, Level 4= could result in death or irreversible, debilitating damage, or major unknowns with respect to the intervention

^b^Protocols reviewed by the IRB prior to 2007 did not have their risk level determined

^c^Clinical department I (Department of Internal Medicine including Oncology, Gastroenterology, Rheumatology, Endocrinology, Nephrology, Cardiology, Hematology, Pulmonology); Clinical department II (Department of Surgery including General surgery, Transplantation, Orthopedic surgery, Cardiovascular surgery, Neurosurgery, Plastic surgery); Clinical department III (Family medicine, Urology, Obstetrics and Gynecology, Pediatrics, Neurology, ophthalmology, Emergency medicine, otorhinolaryngology, Rehabilitation, Psychiatry, Dermatology); Supportive departments (Pathology, Laboratory medicine, Anesthesiology and Pain Medicine, Radiology, Nuclear medicine, Radiation oncology); Basic Science departments (Forensic Science, Microbiology, Physiology, Public health, Preventive medicine, Pharmacology)

### Investigator compliance according to reply rate

**Fig. 2:**
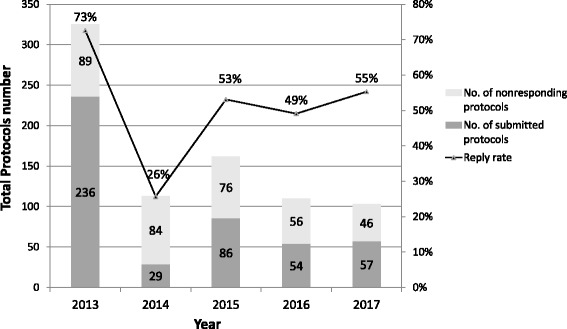
Annual reply rate for screening audits. The screening audit involved a request to the researchers for voluntary submission, and unresponsive researchers could be considered as candidates for subsequent internal audit

The annual reply rates in the screening audits are shown in Fig. 2. Overall, 73% of the investigators submitted TMF for the first screening audit in 2013. Successively, reply rates of 26, 53, 49, and 55% were recorded in the following years. The reason for the large number of candidate protocols in January 2013 was that it was the first screening audit experience, and therefore, included all active protocols approved by the IRB since its establishment. In comparison with the average number of initial reviews in the IRB, approximately 10% are required for screening audit annually.

### Validation of auditor decisions

Since a screening audit primarily focuses on mandatory trial documents, not the content, it is possible to make a relatively objective evaluation. Based on the individual audit checklist, three different auditors gave a grade for the reliability test. The auditors sorted the audit results into four grades (“not a finding,” “minor,” “major”, and “critical”) using the screening audit checklist. The three independent auditors made a decision using the system “Not a finding”=1, “minor” = 2, “major” = 3, and “critical” = 4. The means and standard deviations of the three auditors were 2.46 ± 0.84, 2.35 ± 0.69, and 2.55 ± 0.70, respectively. Weighted kappa values derived from three different pairs of auditors were 0.316 [CI:0.285,0.348], 0.339 [CI:0.305,0.373], and 0.373 [CI:0.342,0.404]), showing fair agreement (weighted kappa of 0.21–0.40) [5].

### Audit outcomes and associated factors

In our study, 55 protocols (11.9%) were “critical,” 180 (39.0%) were “major,” 198 (42.9%) were “minor,” and 29 (6.3%) were “not a finding.” Differences in grade according to year were not statistically significant (*p* = 0.813). Frequencies of protocols determined as “not a finding” and “minor” gradually increased from risk levels 1 to 3 (4.9% → 7.9% → 12.9% and 39.9% → 44.1% → 61.3%, respectively), while frequencies of “major” and “critical” protocols were significantly higher (at 41.1% and 14.1%, respectively) for risk level 1 than risk levels 2 (38.2%, 9.9%) and 3 (22.6%, 3.2%) (*p* = 0.002). International multicenter studies had higher frequencies of “not a finding” than domestic multicenter or single center studies. In contrast, single center studies had higher proportions of “critical” and “major” than domestic multicenter and international multicenter studies (*p* < 0.0001). According to the responsible entity of the study, grade was not statistically different (*p* = 0.625). Also, non-phase clinical research had more “critical” and “major” protocols than phase-specific research that included phase 1, 1/2, 2, 2/3, 3, and 4, as well as Post Market Surveillance and medical-device trials (14.13%, 42.11% > 3.96%, 27.72%) (*p* < 0.0001). Protocols performed by basic science departments had significantly higher frequencies of a “critical” finding as 21.4% than those by clinical departments (Internal Medicine (7.3%), supportive departments (9.9%), Surgical departments (15.6%), *p* = 0.001) (Table 4).

**Table 4:** Factors associated with audit outcomes

	Grading				*p*-value
	Not a finding	Minor	Major	Critical	
Year					
2013	12 (5.1)	104 (44.1)	91 (38.6)	29 (12.3)	0.813^a^
2014	4 (13.8)	11 (37.9)	9 (31.0)	5 (17.2)	
2015	10 (11.6)	30 (34.9)	34 (39.5)	12 (14.0)	
2016	2 (3.7)	27 (50.0)	21 (38.9)	4 (7.4)	
2017	1 (1.8)	26 (45.6)	25 (43.9)	5 (8.8)	
Risk					
Level 1	13 (4.9)	105 (39.9)	108 (41.1)	37 (14.1)	0.002^a^
Level 2	12 (7.9)	67 (44.1)	58 (38.2)	15 (9.9)	
Level 3	4 (12.9)	19 (61.3)	7 (22.6)	1 (3.2)	
Level 4	0 (0)	0 (0)	1 (100)	0 (0)	
Undetermined*	0 (0)	7 (46.7)	6 (40.0)	2 (13.3)	
Multicenter					
Single center	13 (4.63)	108 (38.43)	121 (43.06)	39 (13.88)	< 0.0001^a^
Domestic multicenter	10 (6.17)	83 (51.23)	54 (33.33)	15 (9.26)	
International multicenter	6 (31.58)	7 (36.84)	5 (26.32)	1 (5.26)	
Responsible entity					
Investigator initiated	28 (6.15)	195 (42.86)	177 (38.9)	55 (12.09)	0.6248^b^
Sponsor initiated	1 (14.28)	3 (42.86)	3 (42.86)	0 (0)	
Phase					
Phase1, 1/2, 2, 2/3, 3, 4, PMS, medical-device	11 (10.89)	58 (57.43)	28 (27.72)	4 (3.96)	< 0.0001^a^
Non-phase clinical research	18 (4.99)	140 (38.78)	152 (42.11)	51 (14.13)	
Department					
Clinical department I	17 (9.5)	86 (48.0)	63 (35.2)	13 (7.3)	0.001^a^
Clinical department II	3 (6.7)	18 (40.0)	17 (37.8)	7 (15.6)	
Clinical department III	5 (4.1)	48 (39.0)	48 (39.0)	22 (17.9)	
Supportive departments	4 (4.0)	42 (41.6)	45 (44.6)	10 (9.9)	
Basic Science departments	0 (0)	4 (28.6)	7 (50.0)	3 (21.4)	
Total	29 (6.3)	198 (42.9)	180 (39.0)	55 (11.9)	

*Protocols reviewed by the IRB prior to 2007 did not have their risk level determined

Values are presented as N (%)

^a^Chi-squre test

^b^Fisher’s exact test

### Audit findings

We categorized the audit findings into five types: 1) Failure to maintain essential documents, 2) inappropriateness of documents, 3) failure to obtain informed consent, 4) inappropriateness of informed consent form, and 5) failure to protect participants’ personal information. The findings were collected as numeric counts. The number of findings was related to different types of questions in the screening audit checklist. The severity or proportion of failure to total participants was not reflected. Table 5 shows the association between grades and finding categories. Higher frequencies of “inappropriateness of documents” were prevalent in “not a finding” and “minor” findings (*p* < 0.0001). Also, higher frequencies of “failure to obtain informed consent” and “inappropriateness of informed consent form” were associated with audit result of higher grade (*p* = 0.0001 and *p* < 0.0001, respectively). When auditors found that informed consent was either not obtained or had undergone a false consent process, they tended to regard it as serious non-compliance. In contrast, “failure to maintain essential documents” was not significantly associated with grade (*p* = 0.091).

**Table 5:** Association of grade and five audit finding categories (N=462)

Categories	Grade				*p*-value^a^
	Not a finding	Minor	Major	Critical	
Failure to maintain essential documents	4(19.0)	69(32.5)	78(25.6)	25(21.0)	0.091
Inappropriateness of documents	8(38.1)	87(41.0)	57(18.7)	17(14.3)	<0.0001
Failure to obtain informed consent	0(0.0)	9(4.2)	24(7.9)	21(17.6)	0.0001
Inappropriateness of informed consent form	6(28.6)	29(13.7)	87(28.5)	44(37.0)	<0.0001
Failure to protect participants’ personal information	3(14.3)	18(8.5)	59(19.3)	12(10.1)	0.003

Values are presented as N (%) and these numbers were calculated based on the twenty questions with the screening audit checklist; ^a^ Chi-squre test

### Routine audit as a compliance monitoring method of screening audit

If serious non-compliance was detected in any protocols through a screening audit, they could have been subjected to routine audit. During our study period, 29 protocols received subsequent routine audits due to issues that arose from the screening audit. Specifically, 12 “non-responding” studies and 17 studies determined as “critical” in the screening audit were involved. Two studies were determined as “major,” although the auditors considered it is necessary to perform subsequent monitoring via routine audit. In a compliance monitoring process subsequent to the screening audit, 17.2% of protocols were determined as “critical,” the same as that in the screening audit; 44.8% were determined as “major”; and 37.9% were “minor” in the final IRB decision. Differences between the “critical, major” group and the “non-responding” group were not significant (Table 6).

**Table 6:** Results of routine audit after detection of non-compliance from screening audit

Result from screening audit	Final IRB decision			Total
	Critical (n=10)	Major (n=26)	Minor (n=22)	
Critical, major	3 (17.6)	8 (47.1)	6 (35.3)	17 (100)
Non-responding	2 (16.6)	5 (41.7)	5 (41.7)	12 (100)
Total	5 (17.2)	13 (44.8)	11 (37.9)	29 (100)

Values are presented as N (%); Routine audit was conducted on the protocols that the several non-compliances were found in the screening audit or the non-responded protocols

## Discussion

Laws and regulations related to clinical research have become more stringent in response to the dramatic increase in the number and diversity of clinical research during recent decades [6]. These changes have made QA more important [7]. QA activities intending to improve research quality may involve inspection, sponsor monitoring, and audits of research institutions [8]. Audits are routinely conducted to confirm whether clinical research has been performed in accordance with regulations and the protocol. Audits are a relatively independent and vigorous QA activity; however, they are laborious and time-consuming. The reason for this is that the auditor must be well acquainted with relevant regulations and the study’s procedure. Furthermore, a sufficient training period is needed for the same reason [9]. Thus, the authors developed the novel screening audit as a simple and easy method for HRPPs. The screening audit is executed to grasp the overall management status of clinical research conducted after IRB approval is granted and to confirm the appropriate management of essential documents. It can be conducted using a yes/no checklist, such that auditors without sufficient experience can also be involved, as shown in Table 2.

One of the most important advantages of the screening audit is that it is very easy to adapt to a real-world setting. The checklist consists of 20 questions extracted from a routine audit checklist, with one being that an essential documents list should be maintained. It has no source document or medical record verification or investigator interviews, and it only takes about 30 min to 2 h to complete, which is much shorter than a routine audit. Secondly, a screening audit is an opportunity to inform all investigators about essential trial documents. Because every investigator who is conducting ongoing clinical research is a candidate, it can cover almost every investigator in the institution. With the screening audit, almost every investigator can be informed about clinical research QA activity and basics of study document composition. Furthermore, a routine audit can only detect problems with a randomly chosen protocol, while a screening audit can assess overall research compliance in an institution. In the baseline characteristics of screening audit protocols, phase 1 to 4 clinical trials, post-market surveillance, research with medical devices, and non-phase clinical research are all covered. It was possible for us to observe investigators’ perceptions of QA activity within HRPPs, which have gradually improved, as seen in the annual reply rate. Also, a screening audit can cover the blind spots in a routine audit, such as minimal risk studies or studies conducted by basic science departments.

In recent decades, audits have become more effective in several ways. Califf et al. [10] first provided a cost-effective auditing system in 1997; thereafter, risk analysis has tended to be adopted in research monitoring systems [11]. Most recently, FDA announced guidelines regarding a risk-based approach to monitoring [12]. Risk-based monitoring uses several factors to select protocols, such as the complexity of the study design, the types of endpoint, the clinical complexity of the study population, the relative experience of the investigator, etc. As a result of the screening audit, studies with risk levels 1–2 or conducted by basic science departments or supportive departments were observed as having more “major” or “critical” findings than risk level 3–4 studies or those conducted by departments of internal medicine or surgery. This may indicate the needs for investigator training in basic science departments and supportive departments, as well as low-risk studies.

Previous research assessed U.S. FDA inspection results from 1977 to 2009 and provided the distribution of audit findings for FDA-approved clinical trials [13]. In this article, “failure to follow investigational plan” was frequently observed, at 33.8%. The remaining results were as follows: 28.1% for “inappropriateness of informed consent procedure,” 27.0% for “inappropriateness of documentation,” 15.2% for “insufficient management of investigational product,” and 5.9% for “unreported adverse events.” In our study, “failure to maintain essential documents” was the most frequently observed, while “failure to obtain informed consent forms” was relatively rare. We assumed that the difference had resulted from the screening audit’s focus on documents. However, findings of any misconduct in the informed consent procedure were key factors in determining the grade as “major” or “critical.” Since an appropriate consent procedure is more crucial than minor procedural errors, such as the failure to maintain current versions of study documents in terms of human participant protection [12, 14].

Screening audit can be used as an effective selection tool for routine audit. In this article, we defined investigator non-compliance as “non-responding” and “critical.” Those studies showed high rates of “critical” and “major” grades in the final IRB decision after the internal audit was conducted. “Critical” (17.2%) and “major” (44.8%) grades were relatively frequent for non-compliant studies that needed further routine audit. Taking these findings together, we suggest that “critical” or “non-responding” studies should be regarded as primary candidates for routine audit to ensure a scrupulous review. Furthermore, screening audits are limited when it comes to checking the appropriateness of documentation, which enables an objective evaluation. Auditing is a complex procedure and it changes according to study characteristics [10]. We analyzed the results of audit grading reliability on the same studies by three different auditors, and found a fair degree of agreement.

This study has several limitations. First, it was a retrospective study that was based on records from an audit database. Nevertheless, we were able to develop the concept of screening audits by performing a well-organized, statistical analysis with a grading index. Second, the data were collected from a single tertiary hospital that was running an HRPP. This might limit the generalizability of our data to the general research environment. However, our institution has approximately 1500 ongoing studies, which is highest in Korea, so we believe that the bias would be minimal.

## Conclusion

Screening audits are an effective method for overseeing and controlling an institution’s overall GCP compliance, and can be useful selection criteria for a routine audit. The usefulness of screening audits has not yet been established because of insufficient evidence. However, based on our study, this new screening audit seems to be an effective monitoring method in the challenging environment of clinical research ethics and its oversight, where the increasing variability and magnitude of clinical research make things difficult. Further, multi-center, multi-national studies are needed to validate our conclusion.

## Abbreviations

FDA: Food and Drug Administration
GCP: Good Clinical Practice
HPC: Human research Protection Center
HRPP: Human Research Protection Program
ICF: Informed consent form
IRB: Institutional Review Board
PMS: Post market surveillance
QA: Quality assurance
TMF: Trial master files
